# Supplementary material for: Optimal Treatment of 6-Dimethylaminopurine Enhances the In Vivo Development of Canine Embryos by Rapid Initiation of DNA Synthesis
Source: Int J Mol Sci. 2021 Jul 20;22(14):7757. doi: 10.3390/ijms22147757 (PMC8303139; doi:10.3390/ijms22147757)
Supplement: Supplementary file 1 [file ijms-22-07757-s001.zip › ijms-1298076-supplementary.pdf]

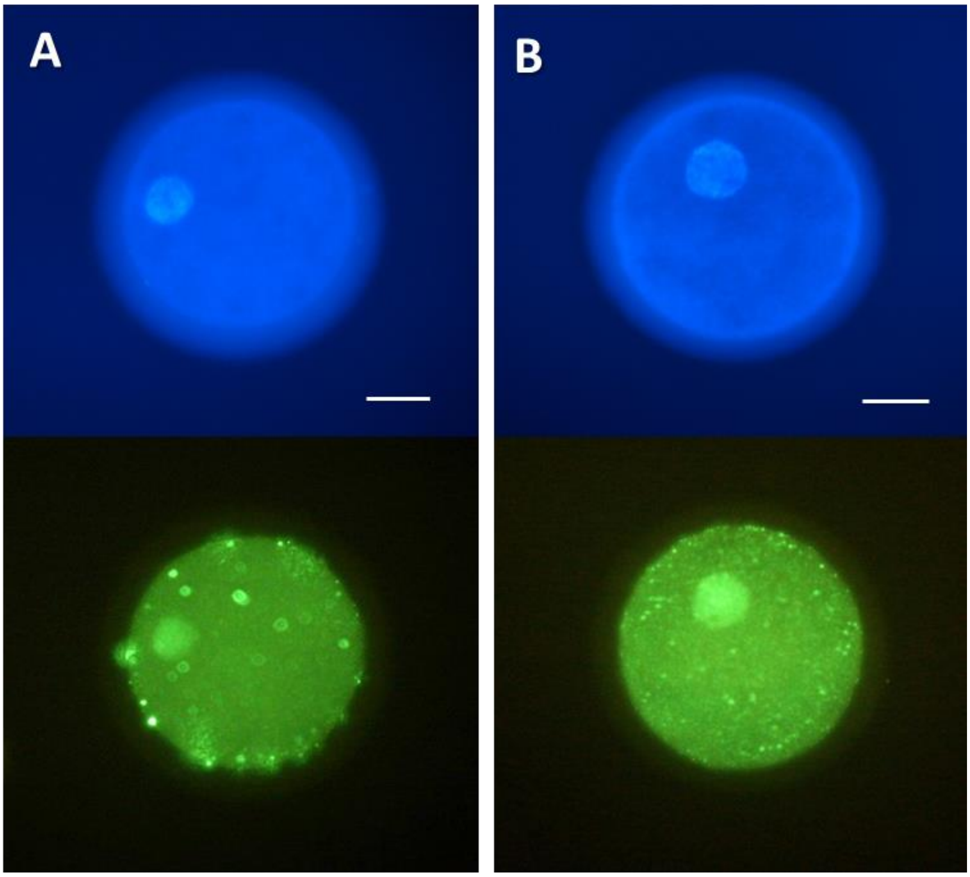

Supplementary Figure S1. BrdU incorporation by canine cloned embryos following oocyte activation. (A) DAPI staining (upper panel) and BrdU incorporation (lower panel) of embryos at 2 hpa in the DMAP-2h group. (B) DAPI staining (upper panel) and BrdU incorporation (lower panel) of embryos at 4 hpa in the DMAP-4h group. (scale bar = 40 mm).
